# The Aqueous Extract of *Polypodium leucotomos* (Fernblock^®^) Regulates Opsin 3 and Prevents Photooxidation of Melanin Precursors on Skin Cells Exposed to Blue Light Emitted from Digital Devices

**DOI:** 10.3390/antiox10030400

**Published:** 2021-03-06

**Authors:** Mikel Portillo, Manuel Mataix, Miguel Alonso-Juarranz, Silvia Lorrio, María Villalba, Azahara Rodríguez-Luna, Salvador González

**Affiliations:** 1 Department of Biology, Faculty of Sciences, Autónoma University of Madrid (UAM), Instituto Ramón y Cajal de Investigación Sanitaria (IRYCIS), 28049 Madrid, Spain; mikel.portillo@uam.es (M.P.); manuel.mataix@estudiante.uam.es (M.M.); silvia.lorrio@gmail.com (S.L.); 2 Maxillofacial Surgery Unit. San Carlos Clinical Hospital, 28040 Madrid, Spain; miguelalonsojuarranz@gmail.es; 3 Medical Affairs Department, Cantabria Labs, 28043 Madrid, Spain; maria.villalba@cantabrialabs.es; 4 Innovation and Development, Cantabria Labs, 28043 Madrid, Spain; 5 Department of Medicine and Medical Specialties, Alcalá de Henares University, 28805 Madrid, Spain

**Keywords:** blue light, melanogenesis, *Polypodium leucotomos*, Opsin-3, photoxidation, hyperpigmentation

## Abstract

The effects of sun exposure on the skin and specifically those related to pigmentation disorders are well known. It has recently been shown that blue light leads to the induction of oxidative stress and long-lasting pigmentation. The protective effect of an aqueous extract of *Polypodium leucotomos* (Fernblock^®^) is known. Our aim was to investigate the action mechanism of Fernblock^®^ against pigmentation induced by blue light from digital devices. Human fibroblasts (HDF) and murine melanocytes (B16-F10) were exposed to artificial blue light (a 400–500 nm LED lamp). Cell viability, mitochondrial morphology, and the expression of the mitogen-activated protein kinase (MAPK) p38, known markers involved in the melanogenesis pathway, were evaluated. The activation of Opsin-3, a membrane protein sensitive to blue light that triggers the activation of the enzyme tyrosinase responsible for melanogenesis in melanocytes, was also analyzed. Our results demonstrated that pretreatment with Fernblock^®^ prevents cell death, alteration of mitochondrial morphology, and phosphorylation of p38 in HDF exposed to blue light. In addition, Fernblock^®^ significantly reduced the activation of Opsin-3 in melanocytes and the photo-oxidation of melanin, preventing its photodegradation. In sum, Fernblock^®^ exerts beneficial effects against the detrimental impact of blue light from digital devices and could prevent early photoaging, while maintaining skin homeostasis.

## 1. Introduction

Skin acts as a physical barrier between internal and external factors, maintaining homeostasis [1]. Exposure of the skin to external factors such as pollution, sunlight, or temperature [2], together with different internal factors [3], has a negative impact on the skin architecture. It is well known that one of the factors that accounts for most of this harmful impact is sunlight exposure [4].

Even though ultraviolet radiation (UV, 100–400 nm) is known to be accountable for most of the skin damage [5], visible light (VIS, 400–700 nm), which represents the vast majority of the terrestrial sunlight after infrared light, has been seen to cause damage by inducing production of free radicals, DNA damage, immunosuppression, and photoaging, among others [6].

The VIS spectrum can be mainly categorized into blue (400–495 nm), green (495–565 nm), yellow (565–590 nm), and red light (590–700 nm) according to the emission wavelength. VIS has extensively been used in medical practice; nevertheless, recent studies suggest that skin cells, specially fibroblasts, are damaged when exposed to this radiation range [7]. Even though red light has been linked to beneficial effects due to its role in enhancing cell growth and synthesis of procollagen I, blue light in contrast has shown to be detrimental for skin homeostasis. Blue light increases the production of reactive oxygen species (ROS), inflammatory mediators, and DNA damage [8], exerts anti-proliferative effects [9], induces melanogenesis activation, decreases collagen production, and alters metalloproteinase activities [10], resulting in early photoaging and hyperpigmentation.

Recent studies have sparked an increased interest in the role of the photooxidation of melanin in hyperpigmentation. Oxidation of preexisting melanin seems to contribute to the increase in pigmentation upon UVA irradiation, and blue light might have the same effect. 5,6-dihydroxyindole-2-carboxylic acid (DHICA)-melanin is oxidized by UVA to form indole-5,6-quinone-2-carboxylic acid (IQCA), which is further degraded to pyrrole-2,3,5-tricarboxylic acid (PTCA), liberated as free PTCA. Studies show how the free/total PTCA ratio serves as a sensitive indicator of the photodegradation of melanin [11].

Furthermore, an additional mechanism that induces melanogenesis has recently been reported. Opsins are light-sensitive G protein-coupled receptors initially described in visual systems, but also present in non-visual systems, such as the epidermis [12]. Opsin-3 (OPN3) has been shown to have strong absorption of the shorter wavelengths of the VIS spectrum [13] and it has been reported to induce melanogenesis when activated by inducing an increase in melanogenesis enzymes such as tyrosinase and dopachrome tautomerase. Therefore, OPN3 acts as a sensor for shorter wavelengths of VIS such as blue light and thus contributes to hyperpigmentation [14].

Blue light can be found in many artificial sources aside from the sun, such as light-emitted diodes (LED) in electronic devices’ screens, and it is well known that our exposure times to these sources have increased considerably over the last decades [15]. In this regard, extensive exposure times during the day to electronic devices plus exposure to natural sunlight, together with the increasing night-time exposure to artificial light, can be considered to be detrimental to the skin.

In light of the above, a hydrophilic natural extract from *Polypodium leucotomos*, Fernblock^®^ (FB), might protect against the harmful effects of blue light irradiation. FB is rich in phenolic compounds, such as cinnamic, ferulic and chlorogenic acids among others, all known to exert antioxidant effects [16]. In vitro and in vivo studies have demonstrated that the photoprotective effects of FB are due to different mechanisms involving inhibition of production of ROS, prevention of DNA damage and lipid peroxidation, inhibition of loss of cell-extracellular matrix adhesion, and prevention of activation of pro-inflammatory factors to name a few [17,18].

This research aimed to investigate the protective effects of FB against significant molecular and cellular changes in normal human dermal fibroblasts (HDF) and mouse melanocytes induced by exposure to artificial blue light. In particular, cell viability, ROS production, mitochondrial damage, induction of pigmentation, melanin oxidation, and regulation of OPN3 expression were observed. Evaluation of these parameters would serve as an accurate demonstration of the protective effects of FB on normal cellular function and prevention of skin photoaging.

## 2. Materials and Methods

### 2.1. Cell Culture

The B16-F10 mouse melanocyte cell line was kindly provided by Dr. Benilde Jiménez Cuenca, Instituto de Investigaciones Biomédicas “Alberto Sols” UAM-CSIC. Human dermal fibroblasts (HDF) were obtained from a skin biopsy by trypsinization with 0.25% Trypsin-Ethylenediaminetetraacetic acid (EDTA). Cells were cultured in Dulbecco’s modified eagle medium (DMEM) supplemented with 10% *(v/v)* fetal bovine serum (FBS), 1% *(v/v)* penicillin G (100 U/mL), and streptomycin (100 µg/mL) (HyClone Laboratories, South Logan, UT, USA). Cells were maintained under standard conditions at 37 °C, 5% humidity, and 5% CO_2_ in an incubator (Heraeus HERAcell, Thermo Scientific, Waltham, MA, USA).

### 2.2. Natural Extract and Cell Treatments

Fernblock^®^ (FB), a controlled hydrophilic extract from the leaves of *Polypodium leucotomos*, was obtained as lyophilized powder from Cantabria Labs, Madrid, Spain. The extract was stored at room temperature and shielded from light following the supplier’s instructions. Stock solutions were prepared at a concentration of 10 mg/mL in distilled water, under agitation at 25–30 °C. This stock solution was diluted in culture medium with 1% FBS to the desired concentrations. Previous studies have tested its efficacy against UV, VIS, and infrared light at concentrations ranging from 0.01 to 10 mg/mL [18,19,20,21]. Based on these studies, we treated cells with 0.1, 0.3, and 0.5 mg/mL of FB for 24 h before irradiation for different experiments.

### 2.3. Cell Irradiation

Melanocytes and fibroblasts were irradiated with artificial blue light with the aim of studying the effects of the high energy visible light emitted by the screens of electronic devices. A narrow-band LED lamp was used as the blue light source, which emits light of 450–465 nm wavelength and 42.05 mW/cm^2^ power (Segainvex, Madrid, Spain). The irradiation doses applied were 25–151 J/cm^2^. Cells were incubated in phenol red-free DMEM containing 1% FBS and directly irradiated from below in order to avoid any possible shielding effects exerted by the culture medium or the treatments. Immediately after irradiation, fresh medium was added, and the cells were maintained in the incubator.

### 2.4. MTT Cell Viability Assay

Cell viability was evaluated 24 h after irradiation using the MTT (3-[4,5-dimethylthiazol-2-yl]-2,5-diphenyltetrazoliumbromide) assay. MTT solution (100 μg/mL) was added to the cell cultures and incubated for 3 h at 37 °C. The resulting precipitate of formazan precipitate was dissolved in dimethylsulfoxide (DMSO, Panreac, Barcelona, Spain) and absorbance was measured at 542 nm using a plate reader (SpectraFluor, Tecan, Zürich, Switzerland). Data were normalized with respect to non-irradiated control values.

### 2.5. Reactive Oxygen Species Measurement

Production of ROS was determined by fluorescence microscopy using the dihydrofluorescein diacetate (DHFDA) fluorescent probe. Cells were incubated with FB for 24 h, loaded with DHFDA (Abcam, Cambridge, UK) to a final concentration of 7.5 M for 50 min at 37 °C and then subjected or not to blue light irradiation. When DHFDA crosses the cell membrane, it is then hydrolyzed by intracellular esterases to its non-fluorescent form, dihydrofluorescein, which reacts with intracellular ROS, resulting in the fluorescent dye fluorescein. After light exposure, cells were immediately observed in the fluorescence microscope.

### 2.6. Mitochondrial Morphology and Membrane Potential

In order to assess mitochondrial morphology, a MitoTracker green fluorescent probe (Invitrogen, Thermo Fisher Scientific, Waltham, MA, USA) was used. After irradiation, cells were incubated with 1 μg/mL of the probe for 15 min at 37 °C. In order to evaluate mitochondrial membrane potential, 1 μg/mL of JC-1 (5,5,6,6′-tetrachloro-1,1′,3,3′-tetraethylbenzimi-dazoylcarbocyanine iodide)green fluorescent dye (Invitrogen) was used for 30 min at 37 °C. JC-1 crosses cell membranes and accumulates inside negatively-charged mitochondria in a potential-dependent manner, shifting its fluorescence emission to red due to the formation of red fluorescent J-aggregates. This way, the red/green fluorescence intensity ratio serves as an indicator of membrane potential, where a decrease in the ratio indicates mitochondrial depolarization. Images were obtained using an epifluorescence microscope with blue and green excitation filters.

### 2.7. Western Blot

Cellular extracts were obtained using Radioimmunoprecipitation Assay (RIPA) buffer with Triton, pH 7.4 (bioWORLD, Dublin, OH, USA), containing protease (complete ULTRA tablets Mini EDTA-free EASYpack, Roche, Mannheim, Germany) and phosphatase (PhosSTOP EASYpack, Roche) inhibitors. Protein concentration was determined using the Pierce BCA Protein Assay Kit (Thermo Fisher Scientific). Protein extracts were diluted in Laemmli buffer (Bio-Rad, Hercules, CA, USA) and heated for 5 min at 98 °C. A Mini-PROTEAN cell was used for electrophoresis in acrylamide/bisacrylamide gels in denaturing conditions (SDS-PAGE). Protein transfer onto polyvinylidene difluoride (PVDF) membranes (Bio-Rad) was then performed with a Transblot Turbo system (Bio-Rad). Membranes were blocked with 5% skimmed milk in 0.1% TBS-Tween 20, and then incubated with primary antibodies anti-p38, anti-phospho-p38 (Cell Signaling Technology, Danvers, MA, USA), and anti-glyceraldehyde-3-phosphate dehydrogenase (GAPDH) (Abcam), and peroxidase-conjugated secondary antibodies (GE Healthcare, Chicago, IL, USA). Protein bands were visualized by chemiluminescence (Pierce ECL Plus Kit, Thermo Fisher Scientific) using a high-resolution image acquisition system (ChemiDoc XRS+, Bio-Rad), and analyzed using Image Lab version 2.0.1 software (Bio-Rad). Data were normalized with respect to the loading control and then to the non-irradiated control values.

### 2.8. Determination of Hyperpigmentation

In order to measure hyperpigmentation, the protocol described by Bellei et al., 2008 [22] was followed, with some modifications. Intracellular and extracellular melanin were measured. Cell culture medium was collected in order to measure extracellular melanin. For intracellular melanin content measurement, cells were first washed with cold PBS and lysed with RIPA buffer with Triton, pH 7.4 (Bioworld), containing protease (complete ULTRA tablets Mini EDTA-free EASYpack, Roche) and phosphatase (PhosSTOP EASYpack, Roche) inhibitors. Lysates were then centrifuged at 17,000× *g* for 10 min at 4 °C. Supernatants were used to measure protein concentrations. The intracellular melanin pigments present in the pellets and the extracellular melanin pigments were solubilized by incubation in 1 M NaOH for 2 h at 60 °C. Absorbance was measured at 405 nm using a plate reader (SpectraFluor, Tecan, Männedorf, Switzerland. For each experiment standard curves were prepared using synthetic melanin (0–250 μg/mL, Sigma). Data were normalized with respect to the protein content and the non-irradiated control.

### 2.9. Microscopic Observation and Quantification

Images were obtained using an epifluorescence microscope (Olympus BX-61) coupled to a DP70 CCD camera equipped with the corresponding filter sets: blue (450–490 nm, exciting filter BP 490) and green (545 nm, exciting filter BP 545). Fluorescence intensity analyses (DHFDA and JC-1 expression) were performed using ImageJ version 1.52a (NIH, Bethesda, MD, USA). Data were normalized with respect to non-irradiated control values.

### 2.10. Quantification of the Expression of Opsin3 by Real-Time Polymerase Chain Reaction

Melanocytes were incubated with FB for 24 h before irradiation. A lower dose of blue light irradiation (25 J/cm^2^) was used for the OPN3 expression levels measurement since extensive cell death was aimed to be avoided. Forty-eight hours after irradiation, cells were washed with PBS 1X, scraped, and centrifuged. Supernatant was discarded and the pellet was stored at −80 °C until RNA extraction and Real-Time Polymerase Chain Reaction (RT-PCR) was performed. mRNA was isolated using a mini RNeasy kit (Qiagen). Concentration and purity of the RNA was determined by spectrophotometry (NanoDropND1000, Nanodrop Technologies). Expression of OPN3 mRNA was evaluated by RT-PCR using the corresponding Taqman probe (Mm00438648_m1, ThermoFisher Scientific). Results were analyzed using the delta-delta-cycle threshold (ddCt) method.

### 2.11. DHICA Oxidation by Blue Light Irradiation

5,6-Dihydroxyindole-2-carboxylic acid (DHICA) was purchased from Santa Cruz Biotechnology. DHICA-melanin solution (1 mg/mL) was prepared from DHICA in PBS 1X under vigorous mixing. To test the protective effect of FB on DHICA-melanin oxidation, the extract was added at final concentrations of 0.3 and 0.5 mg/mL to the DHICA water solutions immediately before irradiation. L-Ascorbic acid (Sigma-Aldrich) was added to DHICA solutions as a protective positive control (due to its well-known antioxidant effect), at 2 mg/mL [11]. DHICA-melanin solutions were subjected to different blue light irradiation doses in 12-well plastic plates. Control of DHICA-melanin oxidation was performed under the same conditions aforementioned using a UVA lamp instead, at a dose of 3.97 mW/cm^2^ [11]. UV/VIS spectra of DHICA-melanin solutions were measured at 0, 5, and 24 h after blue or UVA irradiation. Differential spectra were constructed with absorbance data collected every 2 nm between 250 and 750 nm with a spectrophotometer. The experiment was repeated three times with three points per condition.

### 2.12. Statistical Analysis

Data are represented as the mean ± standard error of the mean (SEM) of at least three independent experiments. For statistical analysis, analysis of variance (ANOVA) and Bonferroni post hoc tests were run using GraphPad Prism 5.00 (GraphPad Software, Inc., San Diego, CA, USA). Differences were considered to be significant when *p* ≤ 0.05.

## 3. Results

### 3.1. FB Prevents Reduction in Cell Viability Induced by Blue Light Exposure in HDF

**Figure 1:**
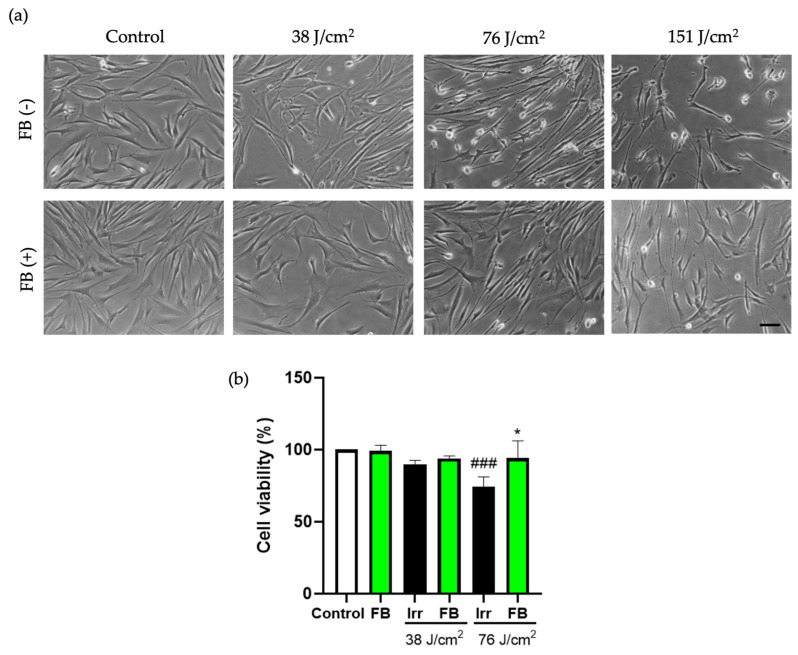
Changes in human dermal fibroblasts (HDF) morphology (**a**) and viability (**b**) at different doses of blue light irradiation and pre-treatments with Fernblock^®^ (FB). Cells were incubated with FB 0.5 mg/mL for 24 h and then irradiated with blue light at different doses (38, 76 and 151 J/cm^2^). Cell viability was evaluated by MTT (3-[4,5-dimethylthiazol-2-yl]-2,5-diphenyltetrazoliumbromide) assay performed 24 h after irradiation (*n* ≥ 3). Data were expressed as % of non-irradiated control cells. Data are shown as mean ± standard error of the mean (SEM). ### *p* < 0.001 vs. control cells and * *p* < 0.05 vs. the corresponding irradiated (Irr) cells. Scale bar: 50 µm.

To study the effect of blue light on cell viability and morphology changes in HDF, different doses of irradiation (38, 76, and 151 J/cm^2^) were tested. The phase contrast images (Figure 1a) showed that as irradiation dose increased, cell viability decreased, and morphology perturbations were detected. Based on these results, we selected the lower dose of blue light (38 J/cm^2^) for oxidative stress, mitochondrial damage, and hyperpigmentation assays. As shown by the MTT assay (Figure 1b), FB 0.5 mg/mL alone not only did not affect cell viability, but induced a protective effect upon irradiation, reaching percentages of cell viability and cell morphology similar to those of unirradiated cells even after 76 J/cm^2^.

### 3.2. Oxidative Stress Induced by Blue Light Irradiation Is Prevented by FB in HDF

**Figure 2:**
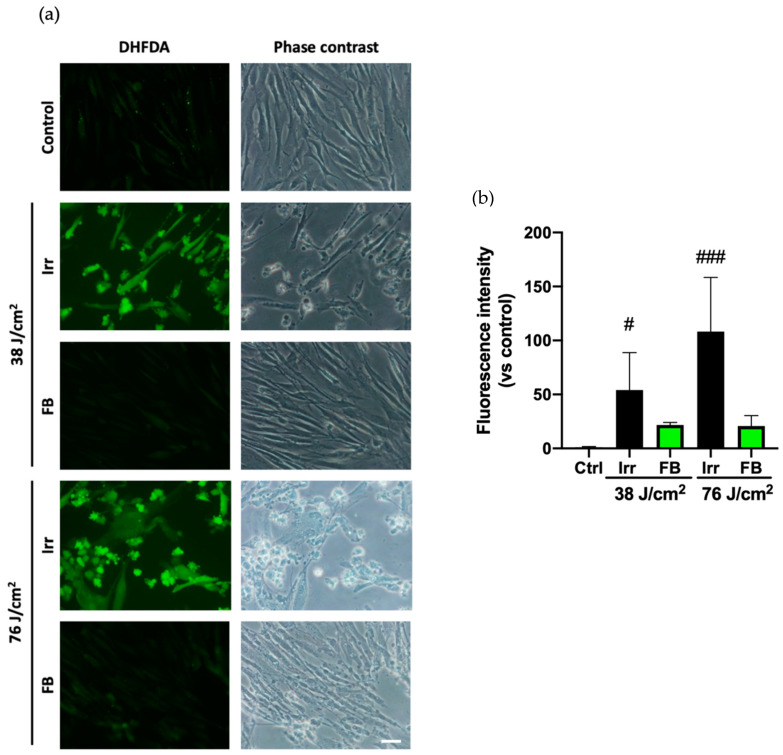
Oxidative stress in HDF exposed to artificial blue light and effect of pre-treatment with FB. Oxidative stress was evaluated by the dihydrofluorescein diacetate (DHFDA) assay. Cells were incubated with FB 0.5 mg/mL for 24 h, loaded with DHFDA, exposed to 38 and 76 J/cm^2^ of blue light radiation, washed, and observed immediately under the microscope (**a**). Quantification of DHFDA fluorescence was measured using ImageJ (*n* = 4). Data are shown as mean ± SEM (**b**). # *p* < 0.05, ### *p* < 0.001 vs. control. Scale bar: 50 µm.

The oxidative stress in HDF exposed to blue light was evaluated using dihydrofluorescein diacetate (DHFDA). The fluorescence images showed that blue light significantly increased intracellular oxidative stress as compared to control cells (Figure 2A). Phase contrast microscopy showed changes in cell morphology, which included deformation of cell membranes and formation of blebs, related to cell death. Pre-treatment with FB 0.5 mg/mL significantly reduced the production of ROS as shown in the images and the quantification of fluorescence intensity and prevented morphology alterations, avoiding blebs formation and consequently, protecting the cell against oxidative stress-induced damage Figure 2B).

### 3.3. Preventive Effects of FB on Mitochondrial Membrane Potential Alterations Induced by Blue Light in HDF

**Figure 3:**
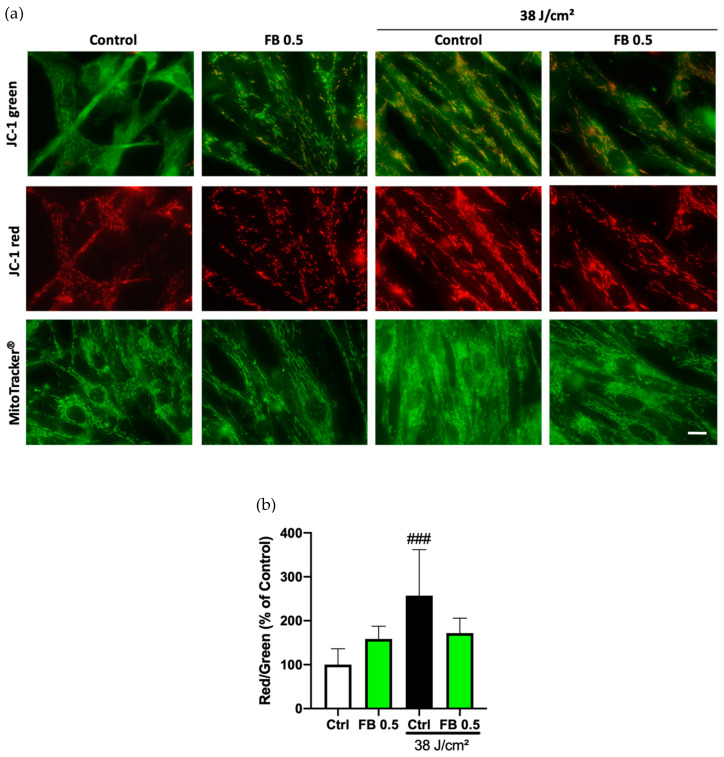
Mitochondrial membrane potential and morphology in HDF exposed to blue light and FB pre-treatment. Changes were evaluated using the mitochondrial potential indicator JC-1 (5,5,6,6′-tetrachloro-1,1′,3,3′-tetraethylbenzimi-dazoylcarbocyanine iodide). Morphology was evaluated using MitoTracker^®^. Non-irradiated and blue light irradiated HDF (**a**). Quantification of JC-1 red/green fluorescence ratio by using ImageJ (**b**). Data are shown as mean ± SEM. ### *p* < 0.001 vs. control. Scale bar 20 µm.

The effect of blue light on the mitochondrial membrane potential of HDF was evaluated using 5,5,6,6′-tetrachloro-1,1′,3,3′-tetraethylbenzimi-dazoylcarbocyanine iodide (JC-1) fluorescent dye. JC-1 red/green fluorescence ratio constitutes an indicator of mitochondrial membrane status. Cells exposed to blue light produced a significant increase in the red/green ratio relative to control cells (Figure 3a), which indicated mitochondrial hyperpolarization (quantified in Figure 3b). However, cells treated with FB before irradiation showed a red/green ratio quite similar to that of the basal status of mitochondrial membrane potential, indicating a preventive effect of FB against hyperpolarization.

### 3.4. FB Regulation of Blue Light-Induced Pigmentation in HDF. Modulation of p38 Phosphorylation

**Figure 4:**
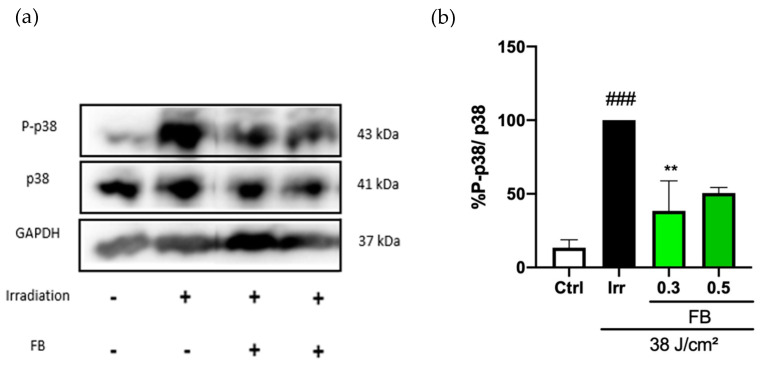
Phosphorylation of p38 in HDF exposed to artificial blue light. Phosphorylation of p38 was evaluated by Western blot (*n* = 3). Cells were incubated in phenol red-free Dulbecco’s modified eagle medium (DMEM) 0% fetal bovine serum (FBS), treated for 24 h with FB (concentrations), irradiated with blue light (38 J/cm^2^) and proteins extracted 1 h post-irradiation. Representative immunoblots (**a**) and their quantification plot (**b**) are shown. Data are expressed as % of non-irradiated cells. Data are shown as mean ± SEM. ### *p* < 0.001 vs control cells and ** *p* < 0.01 vs the corresponding irradiated (Irr) cells.

Phosphorylation of the mitogen-activated protein kinase (MAPK) p38 related to the p38 melanogenic signaling pathway was studied by Western blot in order to evaluate possible alterations caused by blue light. It is known that phosphorylation of p38 leads to the subsequent phosphorylation of other factors, including the melanocyte inducing transcription factor (MITF), which up-regulates the expression of melanogenic enzymes (such as tyrosinase and dopachrome tautomerase) and, therefore, induces an increase in melanin production. After blue light irradiation, we observed an increase in the phosphorylation of p38, which was significantly reduced by the FB pretreatment 1 h after light exposure (especially at a concentration of 0.3 mg/mL), as seen in the immunoblots and plots in Figure 4.

### 3.5. FB Prevents the Hyperpigmentation Induced by Blue Light in Melanocytes

**Figure 5:**
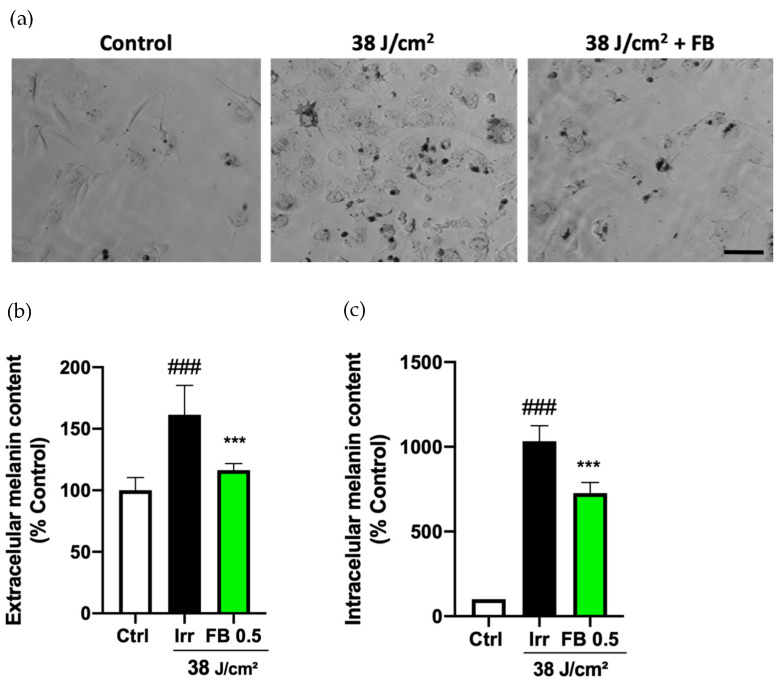
Melanin content in melanocytes exposed to artificial blue light with and without FB pre-treatment (0.5 mg/mL). Microphotographs show how unirradiated (control), irradiated and treated melanocytes produce different amounts of melanin dark granules (**a**). Cells were incubated in phenol red-free DMEM 1% FBS and FB for 24 h, irradiated (38 J/cm^2^), fresh medium replaced, melanin pigments collected, solubilized, and quantified (**b**), and (**c**) 3 h later by absorbance measurements. Data were normalized by mg protein and expressed as % of control cells (n ≥ 4). Data are shown as mean ± SEM. ### *p* < 0.001 vs. control; *** *p* < 0.001 vs. irradiated. Scale bar: 50 µm.

In order to study hyperpigmentation in melanocytes exposed to artificial blue light, production of intracellular and extracellular melanin pigment was measured. Contrast phase images show how blue light irradiation, at the doses employed in HDF, induced hyperpigmentation in melanocytes, visible as dark deposits (granules) in the cultures (Figure 5a). When melanocytes were treated with FB (0.5 mg/mL) prior to blue light exposure, however, intracellular and extracellular melanin production was significantly reduced, as observed in the photographs and melanin content plots (Figure 5b,c). 

### 3.6. FB Prevents the Increase of OPN3 Expression Induced by Blue Light in Melanocytes

**Figure 6:**
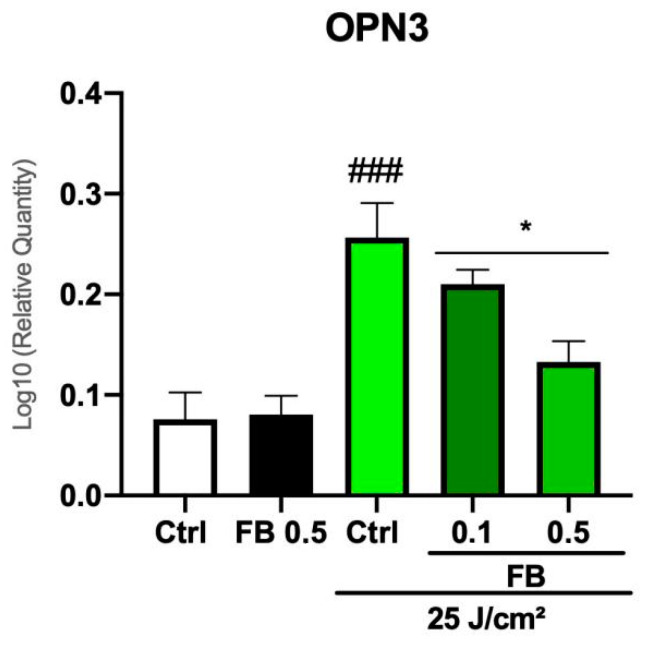
Opsin-3 (OPN3) expression in melanocytes exposed to artificial blue light and the treatments. Data show different expression levels of OPN3 in control, irradiated, and FB-treated melanocytes. Cells were incubated in phenol red-free DMEM 1% FBS with FB for 24 h before irradiation (25 J/cm^2^) and OPN3 expression was quantified 48 h after by RT-PCR. Data are shown as mean ± SEM. ^###^ *p* < 0.001 vs. control; * *p* < 0.1 vs. irradiated.

OPN3 expression levels were quantified in order to evaluate whether blue light irradiation increases its expression. OPN3 acts as a VIS photosensor, activating melanogenic pathways and therefore inducing hyperpigmentation. OPN3 expression levels were quantified by RT-PCR 48 h after blue light irradiation (Figure 6). Results showed that cells increased expression of this photoreceptor after blue light irradiation, whereas FB diminished such expression, providing values similar to those of the controls, especially at the concentration of 0.5 mg/mL.

### 3.7. FB Reduces Spectral Changes Induced by Blue Light in DHICA-Melanin

**Figure 7:**
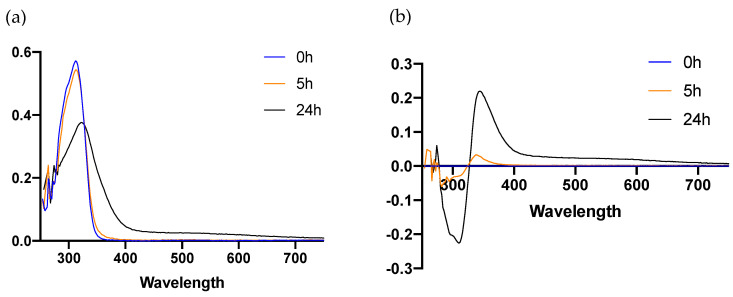
UV-VIS spectra of 5,6-dihydroxyindole-2-carboxylic acid (DHICA)-melanin solutions 24 h after blue light irradiation. Data show changes in the UV-VIS spectrum induced by blue light irradiation over 24 h (**a**). Changes in differential spectrum relative to the 0 h control (**b**). DHICA-melanin solutions UV-VIS spectra were measured before blue light exposure and 5 h and 24 h after irradiation (76 J/cm^2^).

In order to evaluate melanin photooxidation and hyperpigmentation, we analyzed spectral changes in DHICA-melanin solutions induced by blue light. DHICA-melanin solutions are initially characterized by a clear/pale gray color. Upon irradiation with blue light (76 J/cm^2^), they progressively darkened to eventually become brownish 24 h (see supplementary Figure S1). The DHICA-melanin UV/VIS spectrum showed an evident strong absorption maximum at 320 nm (Figure 7a). Differential spectra, taken in relation to the 0 h control, showed a time-dependent decrease in absorbance at 320 nm with an increase at 350 and 550 nm (Figure 7b). The absorbance at 320 nm can be ascribed to DHICA [23], while the absorbance peaks at 350 and 580 nm are similar to those of indole-5,6-quinone-2-carboxylic acid (IQCA) [24]. Irradiation with UVA light was used as a control of DHICA-melanin oxidation (Supplementary Figure S2).

**Figure 8:**
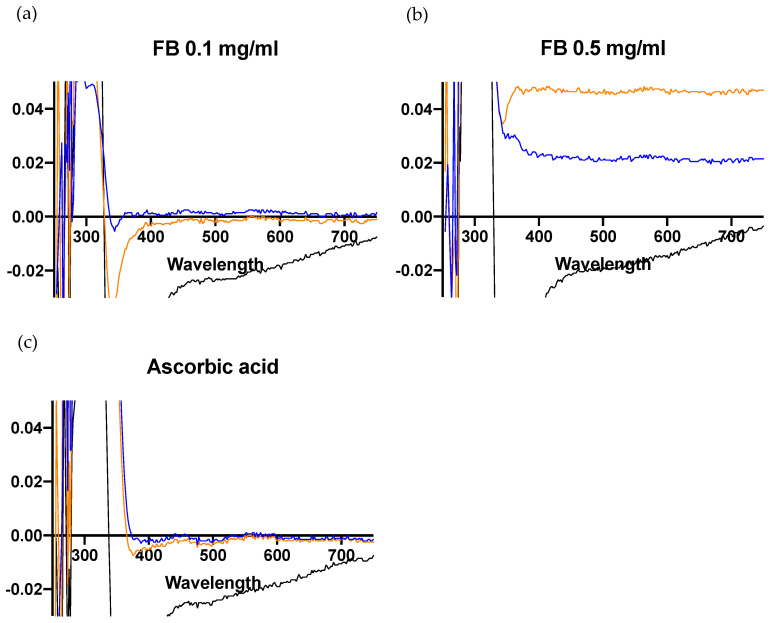
Effects of FB (**a,b**) and ascorbic acid (**c**) on the UV-VIS spectrum. Data show the effects of the different antioxidant agents 5 h and 24 h after blue light irradiation. Differential spectra were taken against the controls without the treatments.

Spectral changes were also examined for the effects of FB (0.1 and 0.5 mg/mL) (Figure 8a,b), and ascorbic acid (2 mg/mL) was used as a control due to its well-known antioxidant effects (Figure 8c). FB demonstrated a protective effect against DHICA-melanin oxidation, as did ascorbic acid, as evidenced by the decrease in absorption at 350 and 580 nm.

**Figure 9:**
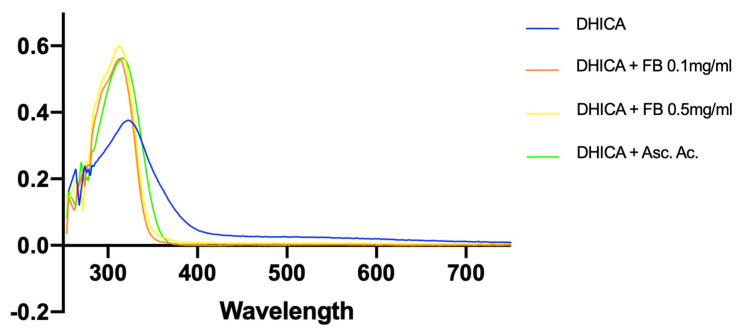
Effects of different antioxidant agents on the UV-VIS spectra of DHICA-melanin solutions 24 h after blue light irradiation. Data show the UV-VIS spectra of DHICA-melanin solutions treated with different antioxidant agents 24 h after blue light irradiation. DHICA-melanin solutions were irradiated together with FB (0.1 and 0.5 mg/mL) and ascorbic acid (2 mg/mL), and UV-visible spectra were measured 24 h after irradiation.

These effects were better observed in Figure 9. A substantial restraint in the decrease at the 320 nm absorption peak (DHICA-melanin) at 24 h can be noted when FB or ascorbic acid were added, which can be ascribed to their antioxidant effects, preventing the oxidation of DHICA-melanin.

## 4. Discussion

The amount of time that people spend in front of digital devices has greatly increased as the population has become progressively digitized and these devices have ultimately become vital for the operability of society. Different studies have demonstrated the undeniable negative effects of these new habits with respect to the development of different psychological and metabolic disorders, and recently an increased interest has arisen regarding their effects on skin homeostasis. Although UV is the main radiation responsible for most of the skin-related adverse effects of sunlight, VIS has also been shown to cause cell damage at different levels. In fact, VIS has been linked to premature skin aging due to free radical production and hyperpigmentation, among other effects [6].

Although there is evidence demonstrating the negative clinical effect of blue light on the development of hyperpigmentation, it is not clear that the use of digital devices exacerbates these conditions. Melanin plays a crucial role in providing protection to the skin against the harmful effects of UV [25], however it is not well known in the case of blue light. Preclinical studies have shown that artificial blue light significantly decreased cell viability in a dose dependent manner in HDF, as discussed by Magni et al. [26], and caused other indirect detrimental effects including formation of cyclobutane pyrimidine dimers [27].

This present work was designed to simulate chronic exposure to blue light emitted by digital devices on HDF and melanocytes in vitro by means of a blue light source with a 42.05 µW/cm^2^ irradiance [28]. The photoprotection activity of a hydrophilic botanical extract from *Polypodium leucotomos*, FB, against potential blue light-induced skin damage was also evaluated. FB is an interesting candidate for providing protection against the previously-stated alterations due to its antioxidant and anti-inflammatory properties [29]. It has been shown to exert an effect on preventing immunosuppression and DNA damage induced by UV and VIS and reducing inflammation, among others [17]. Furthermore, FB has been shown to have anti-tumoral effects in the skin by activating tumor suppressor p53, decreasing UV-induced cell proliferation, and inhibiting UV-induced NF-κB and cyclooxygenase-2 (COX-2) expression, markers deregulated in skin and other epithelial cancers.

The results obtained in this study demonstrate that the blue light radiation dose used, which simulated chronic exposure to digital devices, decreased cell viability and altered physiological cell morphology; these effects were reversed by FB. We also demonstrated that FB prevented the increase of oxidative stress and the mitochondrial damage induced by artificial blue light irradiation. In this regard, the mitochondrial status, as indicated by JC-1 fluorescent dye, showed how blue light changed mitochondrial membrane potential, inducing hyperpolarization. Pre-treatment with FB made the cells recover basal status of mitochondrial membrane potential.

In terms of pigmentation, an increase in the expression of the photoreceptor OPN3 was noted after blue light irradiation, which has been linked to increased melanogenesis as well. After stimulation of OPN3 by blue light, a calcium flux is induced that activates different proteins including extracellular signal-regulated kinase (ERK) and p38, leading to the phosphorylation of MITF, increased tyrosinase, and ultimately, an increase of melanin in cells [14]. OPN3 expression levels were significantly reduced by pre-treatment with FB, suggesting that this photoreceptor plays an important role in melanogenesis activation by blue light. The mechanisms by which FB modulates the expression of OPN3 need to be further investigated. In any case, the demonstration of the pathway that induces melanogenesis after VIS irradiation provides potential targets to prevent this type of hyperpigmentation.

In addition, we observed how artificial blue light induced signal transduction in HDF implicated in melanin production. Different studies have shown that there is a dynamic cross-talk between fibroblasts and melanocytes regarding the MAPK pathway involvement in melanogenesis, where the activation of this pathway consequently leads to the expression of melanogenic enzymes [30]. The activation of the p38 melanogenic pathway by phosphorylation of p38 was observed in our cultures after blue light radiation, as well as an increase in both extracellular and intracellular melanin. These effects were also prevented by FB. We observed that FB reduced phosphorylation of the p38 MAPK pathway, which has been indicated as leading to a reduction in MITF proteins. Activation of the MAPK p38 melanogenic pathway was also reduced by the decrease in p38 phosphorylation. Previous studies have shown how inhibition of p38 phosphorylation induces a decrease in the expression of tyrosinase and melanin in murine melanocytes [31].

In addition, previous studies [11,32] have shown that high doses of UVA radiation can lead to photooxidation of melanin and thus hyperpigmentation. Our study has shown how shorter wavelength VIS is also capable of triggering melanin photooxidation and promoting darkening of melanosomes, contributing to the overall development of hyperpigmentation. Our group previously studied the effect of FB on melanin production but no differences were observed in tyrosinase activity, thus we decided to focus on the antioxidant mechanisms in terms of photooxidation [29].

In this regard, eumelanin photoprotects pigmented tissues from UV damage. It has been demonstrated that UVA-induced tanning seems to result from the photooxidation of preexisting melanin (IQCA/PTCA) not contributing to photoprotection. Results previously published [11] from in vitro studies by using UVA radiation suggest that this radiation induces the conversion of DHICA to IQCA in eumelanin, which then reacts with different ROS to form reactive intermediates. As result of these oxidative processes, eumelanin is photodegraded and PTCA is liberated. Thus, the UVA-induced oxidation of DHICA-melanin is divided into two distinct but continuous stages: oxidation to IQCA and degradation to free PTCA. Our results confirm that FB prevents melanin oxidation induced by blue light and thus reduces pigment darkening.

## 5. Conclusions

In brief, these results highlight the potential beneficial effects of FB in reducing the detrimental impact of increasing exposure to blue light from digital devices by preventing early photoaging and maintaining skin homeostasis. In regards to pigmentation, our results demonstrated that FB prevents it, at least, by several mechanisms: modulation of p38 melanogenic signaling pathway, inhibition of photooxidation of melanin precursors, and reduction of OPN3 expression.

## Data Availability

Not applicable.

## Supplementary Materials

The following are available online at https://www.mdpi.com/2076-3921/10/3/400/s1, Figure S1: Changes in color of DHICA-melanin solutions 24 h after blue light irradiation, Figure S2: UV-VIS spectra of DHICA-melanin solutions 24 h after UVA.
