# Supplementary material for: The Aqueous Extract of Polypodium leucotomos (Fernblock®) Regulates Opsin 3 and Prevents Photooxidation of Melanin Precursors on Skin Cells Exposed to Blue Light Emitted from Digital Devices
Source: Antioxidants (Basel). 2021 Mar 6;10(3):400. doi: 10.3390/antiox10030400 (PMC7998284; doi:10.3390/antiox10030400)
Supplement: Supplementary file 1 [file antioxidants-10-00400-s001.pdf]

Article

The Aqueous Extract of *Polypodium leucotomos* (Fernblock®) Regulates Opsin 3 and Prevents Photooxidation of Melanin on Skin Cells Exposed to Blue Light Emitted from Digital Devices.

Supplementary Materials: The following are available online at www.mdpi.com/xxx/s1, Figure S1: Changes in color of DHICA-melanin solutions 24 h after blue light irradiation, Figure S2: UV-VIS spectra of DHICA-melanin solutions 24 h after UVA irradiation.

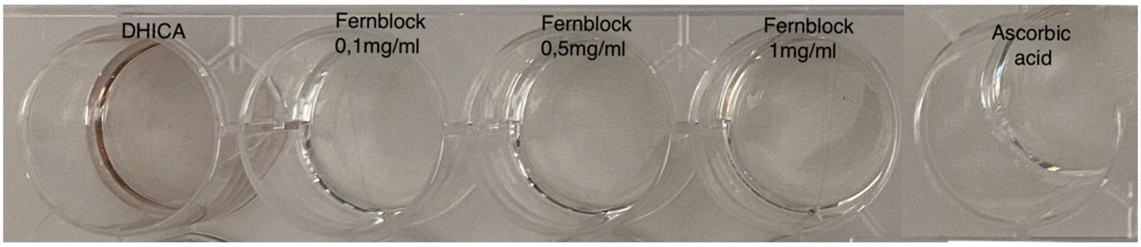

Supplementary figure 1: Changes in color of DHICA-melanin solutions 24 h after blue light irradiation. Image shows the color of DHICA-melanin solutions 24 h after irradiation with blue light (76 J/cm^2). It can be observed how FB and ascorbic acid (used as an antioxidant control) prevent the oxidation, and therefore browning, of the DHICA-melanin solutions.

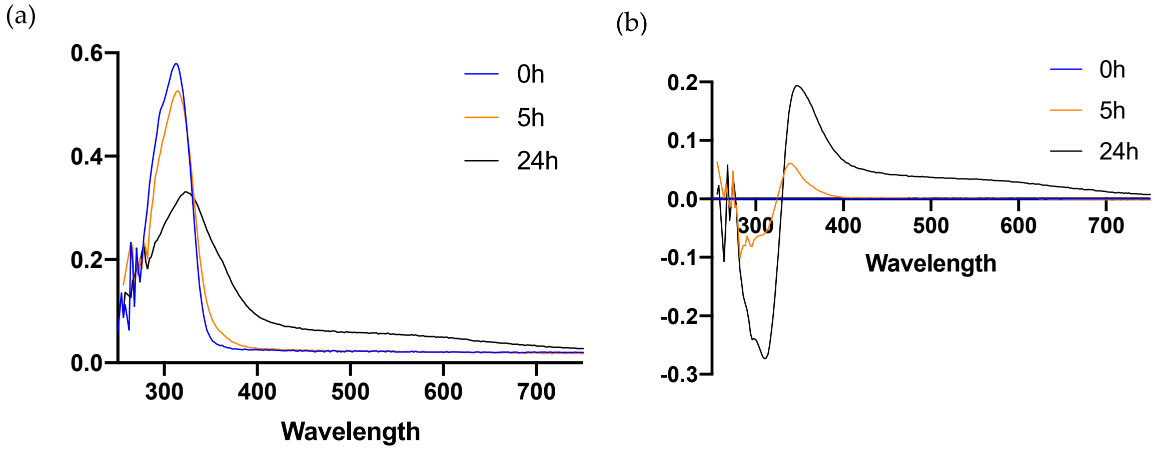

Supplementary figure 2: UV-VIS spectra of DHICA-melanin solutions 24 h after UVA irradiation. Data show changes in the UV-VIS spectrum induced by UVA irradiation throughout 24 h (a). Changes in differential spectrum relative to the 0 h control (b). UV-VIS spectra of DHICA-melanin solutions were measured before UVA exposure and 5 h and 24 h after irradiation.
